# Supplementary material for: Forecasting Thailand’s mobility trends using Feature Engineered XGBoost for pandemic crisis movement management
Source: PLoS One. 2026 Mar 26;21(3):e0345547. doi: 10.1371/journal.pone.0345547 (PMC13020994; doi:10.1371/journal.pone.0345547)
Supplement: Attachment — Submitted filename: Response to Reviewers.docx [file pone.0345547.s009.docx]

Part 1 : Journal Requirement

1. Please ensure that your manuscript meets PLOS ONE's style requirements, including those for file naming. The PLOS ONE style templates can be found at
   <https://journals.plos.org/plosone/s/file?id=wjVg/PLOSOne_formatting_sample_main_body.pdf> and

<https://journals.plos.org/plosone/s/file?id=ba62/PLOSOne_formatting_sample_title_authors_affiliations.pdf>

Response :

Yes, we have made our manuscript complied with the journal’s requirements by changing the style of naming the figure.

2. In your Methods section, please include additional information about your dataset and ensure that you have included a statement specifying whether the collection and analysis method complied with the terms and conditions for the source of the data.

Response :

Yes, we have added a statement specified that the collection and analysis method complied with the terms and conditions of the source of data as follows:

line 76 (new) Datasets declaration statement: In this study, the data collection and analysis method complied with the terms and conditions of the source of the data.

For the information about the dataset, we have already included information about the dataset in the in-text statement which also referred to the references as follows:

line 78 (new): … we use daily mobility data in Thailand retrieved from the Google Community Mobility Reports or GCMR [7],...

line 100 (new) … COVID-19 cases per million people in Thailand retrieved from the COVID-19 data repository maintained by the Johns Hopkins University Center for Systems Science and Engineering or JHU-CSSE [22]

3. Please note that PLOS One has specific guidelines on code sharing for submissions in which author-generated code underpins the findings in the manuscript. In these cases, we expect all author-generated code to be made available without restrictions upon publication of the work. Please review our guidelines at https://journals.plos.org/plosone/s/materials-and-software-sharing#loc-sharing-code and ensure that your code is shared in a way that follows best practice and facilitates reproducibility and reuse

Response :

Yes, we have provided all our generated code available without restrictions upon publication of the work via GitHub at the following address [https://github.com/apisitgo/COVID19-Mobility-Forecasting-TH](https://github.com/apisitgo/COVID19-Mobility-Forecasting-TH/tree/main).

4. Thank you for stating the following financial disclosure:

“This research was supported by the Government-wide R&D to Advance Infectious Disease Prevention and Control, Republic of Korea (grant number: RS-2023-KH140419).

This research was also co-funding by the Faculty of Informatics, Burapha University, Thailand.”

Please state what role the funders took in the study. If the funders had no role, please state: "The funders had no role in study design, data collection and analysis, decision to publish, or preparation of the manuscript."

If this statement is not correct you must amend it as needed.

Please include this amended Role of Funder statement in your cover letter; we will change the online submission form on your behalf.

Response :

We have included the following statements at the end part of our revised cover letter.

“This research was supported by the Government-wide R&D to Advance Infectious Disease
 Prevention and Control, Republic of Korea (grant number: RS-2023-KH140419).

This research was also co-funding by the Faculty of Informatics, Burapha University, Thailand.”

"The funders had no role in study design, data collection and analysis, decision to publish, or preparation of the manuscript."

5. Please include captions for your Supporting Information files at the end of your manuscript, and update any in-text citations to match accordingly. Please see our Supporting Information guidelines for more information: <http://journals.plos.org/plosone/s/supporting-information>.

Response :

We did not provide any supporting information files. So for this issue, we have made no change.

6. If the reviewer comments include a recommendation to cite specific previously published works, please review and evaluate these publications to determine whether they are relevant and should be cited. There is no requirement to cite these works unless the editor has indicated otherwise.

Response :

The reviewer does not comment to include any specific published works. So we did not include any more specific works.

Part 2 : Reviewers' comments

1. *The methodology is well-structured and follows a logical progression from data acquisition to model evaluation.*

*The use of three distinct, high-quality datasets—the Google Community Mobility Reports, the Oxford Stringency Index, and JHU-CSSE epidemiological data—provides a comprehensive view of Thailand's pandemic dynamics. Furthermore, integrating these via a "Date" primary key ensures temporal consistency.
Regarding the model validation, the paper employs a 90:10 train-test split. While the authors justify this as necessary to capture mobility patterns during rapid policy changes, a fixed split in time-series data is often problematic. I recommend implementing time-series cross-validation (such as a rolling origin approach) to demonstrate model stability across different pandemic waves.*

Response :

Thank you for your comment. For this comment, we have added a graph depicting the analysis of MAE of the validation results of each forecasting approach using a Rolling-Origin Evaluation (ROE) method in addition to a 90:10 train-test split. We have categorized the validation results into four event waves, as shown below. We hope this will help demonstrate the stability of our proposed method across different pandemic waves.

*-------------------------------------------------------------------------------------------------------------------------------------*

In order to ensure the highest level of predictive accuracy and model stability, this study employed a dual-validation framework. As discussed earlier, the dataset was evaluated using a standard 90:10 training-to-testing ratio to establish baseline performance. Subsequently, a rigorous Rolling-Origin Evaluation (ROE) strategy was applied to all three forecasting models: ARIMA, Prophet, and Feature Engineered XGBoost (the proposed method). The ROE approach, as illustrated in Figure 7, utilizes an expanding window technique where the forecast origin shifts chronologically across four distinct pandemic phases in Thailand: the Wuhan (Wave 1), Alpha-Beta (Wave 2), Delta (Wave 3), and Omicron (Wave 4) waves.

**Figure 8. Comparison of MAE across 4 COVID-19-Waves** using ARIMA, FB-PROPHET and XGBoost **based on Rolling-Origin Evaluation Approach**

Figure 8 compares MAE values across six mobility categories, namely Retail and Recreation, Grocery and Pharmacy, Parks, Transit Stations, Workplaces, and Residential, over four COVID-19 waves in Thailand using ARIMA, FB-PROPHET and Xgboost based on Rolling-Origin Evaluation Approach. Overall, the Feature Engineered XGBoost model consistently achieves the lowest prediction errors across most mobility categories and pandemic phases, particularly for mobility types that are highly sensitive to policy interventions such as Transit Stations, Workplaces, and Residential areas. During the first wave (Wuhan), prediction errors remain relatively moderate across all models, reflecting more uniform mobility restrictions. More pronounced performance differences emerge in subsequent waves as mobility behavior becomes increasingly heterogeneous. In the Alpha-Beta and Delta waves, MAE values increase notably, especially for Parks and Workplaces, indicating heightened volatility and reduced predictability during periods of rapid and stringent policy changes. In contrast, the Omicron wave exhibits lower MAE values in several categories, suggesting partial behavioral adaptation and stabilization despite ongoing policy adjustments. ARIMA performs reasonably well in mobility categories with smoother trends but shows reduced accuracy during highly variable periods, while Prophet records the highest MAE in most categories, particularly for Parks and Workplaces.

2. *Comparing a traditional statistical model (ARIMA), a trend-based model (Prophet), and a machine learning model (XGBoost) provides a robust benchmark. However, the nomenclature "X-XGBoost" requires clarification. In machine learning literature, "Extended" often implies an algorithmic modification. The authors should specify whether "X-XGBoost" involves an architectural change or simply refers to a standard XGBRegressor with a specific feature set. If the model relies solely on feature engineering, renaming it "Feature-Engineered XGBoost" would prevent confusion regarding potential algorithmic extensions.*

Response :

Thank you for your comment. We have changed X-XGBoost to Feature Engineered XGBoost and also made change to our title too.

3. While the methods effectively tie to the results, some gaps remain. For instance, although "feature importance" (weight and gain) is mentioned in the methods, these results are only briefly summarized. A detailed table or plot showing which features (e.g., Stringency Index vs. Day of Week) were most influential for each of the six mobility categories would better justify the "X-XGBoost" approach.

Response :

Thank you for your comment. For this comment, we have added additional analysis using bar-graph to depict the influencing level to the XGBoost model of each feature important and also added the explanation as shown below. Hope, this could help reader to see the significant of each feature better.

*-------------------------------------------------------------------------------------------------------------------------------------*

In addition to the tabular form of information about important features, we have also conducted an analysis using a bar graph, as illustrated in Figure 9, to depict the influencing level of each important feature. This graph shows the frequency of feature usage in trees (weight), the contribution to loss reduction (gain), and the relative number of samples affected (cover), as shown below.

**Figure 9. Feature importance analysis of the Feature Engineered XGBoost Approach**

Figure 9 illustrates the feature importance analysis of the Feature Engineered XGBoost model for population mobility forecasting across all mobility categories, including Retail and Recreation, Grocery and Pharmacy, Parks, Transit Stations, Workplaces, and Residential. The analysis is evaluated using three complementary importance metrics: Weight, Gain, and Cover, which respectively represent the frequency of feature usage in tree construction, the contribution of each feature to loss reduction, and the proportion of samples affected by feature-based splits.

In the Weight dimension (top panel), which reflects how frequently features are selected during tree construction, avg_all_mobility and temporal features such as day, week, and month consistently exhibit the highest values across all mobility categories. This indicates that historical mobility patterns and temporal structures form the core foundation of the forecasting process. The StringencyIndex plays a secondary but notable role, particularly in policy-sensitive categories such as Workplaces and Transit Stations. In contrast, policy-specific variables such as lockdown_level and is_holiday are used less frequently, suggesting that they function primarily as complementary rather than dominant predictors.

The Gain dimension (middle panel), which measures each feature’s contribution to reducing prediction error, reveals clear structural differences across mobility categories. The feature avg_all_mobility provides substantial gain for Retail and Recreation, Parks, and Transit Stations, while year shows a pronounced influence in Grocery and Pharmacy and Residential mobility, reflecting longer-term structural changes across pandemic periods. Additionally, is_holiday and lockdown_level demonstrate relatively high gain in specific categories such as Workplaces and Transit Stations, indicating strong but localized effects associated with policy interventions or special periods.

In the Cover dimension (bottom panel), which represents the relative proportion of samples influenced by feature splits, temporal variables including avg_all_mobility, year, week, and day consistently affect a large share of observations across all mobility categories. The StringencyIndex and lockdown_level exhibit moderate coverage, particularly in work and travel related categories. Notably, day_of_week shows high coverage in Parks and Transit Stations, highlighting systematic differences in mobility behavior between weekdays and weekends.

4. Finally, to enhance transparency and reproducibility of the machine learning implementation, I strongly recommend that the authors provide the full code or scripts used for data preprocessing, model training, and evaluation. Making these scripts available—either as supplementary material or via a public repository (e.g., GitHub)—would allow other researchers to replicate the results and verify the methodology

Response :

Thank you for your comments, which help make our work beneficial to other researchers and increase the transparency and reproducibility of our work. In this regard, we have made our full code, data preprocessing, model training and evaluation available on GitHub at the following address: [https://github.com/apisitgo/COVID19-Mobility-Forecasting-TH](https://github.com/apisitgo/COVID19-Mobility-Forecasting-TH/tree/main)
