# Supplementary material for: Forecasting Thailand’s mobility trends using Feature Engineered XGBoost for pandemic crisis movement management
Source: PLoS One. 2026 Mar 26;21(3):e0345547. doi: 10.1371/journal.pone.0345547 (PMC13020994; doi:10.1371/journal.pone.0345547)
Supplement: Attachment — Submitted filename: Response to Reviewers_Forecasting_Thailand_6Mar2026.docx [file pone.0345547.s010.docx]

Part 1 : Journal Requirement:

1. If the reviewer comments include a recommendation to cite specific previously published works, please review and evaluate these publications to determine whether they are relevant and should be cited. There is no requirement to cite these works unless the editor has indicated otherwise.

Response :

Noted with thanks. However, there is none of this kind of recommendation.

2. Please review your reference list to ensure that it is complete and correct. If you have cited papers that have been retracted, please include the rationale for doing so in the manuscript text, or remove these references and replace them with relevant current references. Any changes to the reference list should be mentioned in the rebuttal letter that accompanies your revised manuscript. If you need to cite a retracted article, indicate the article’s retracted status in the References list and also include a citation and full reference for the retraction notice.

Response :

Noted with thanks. We have already review reference list as follows:

1. To improve source quality, non-scholarly/tutorial sources were replaced with peer-reviewed primary references:

1.1 Reference [24] was replaced with: Bergmeir C, Hyndman RJ, Koo B. A note on the validity of cross-validation for evaluating autoregressive time series prediction. Comput Stat Data Anal. 2018;120:70–83. https://doi.org/10.1016/j.csda.2017.11.003

1.2 Reference [25] was replaced with: Chen T, Guestrin C. XGBoost: A scalable tree boosting system. Proc 22nd ACM SIGKDD Int Conf Knowledge Discovery and Data Mining. 2016:785–794. https://doi.org/10.1145/2939672.2939785

2. Those non-scholarly and duplicate references were removed:

2.1 Former Reference [32] has been removed.

2.2 Former Reference [33] has been removed

3. The in-text citations in Section 3 (Results and Discussion) were updated accordingly, with former references [32, 33] has been replaced with [24, 25].

Part 2 : Response to Reviewers:

1. If the authors have adequately addressed your comments raised in a previous round of review and you feel that this manuscript is now acceptable for publication, you may indicate that here to bypass the “Comments to the Author” section, enter your conflict of interest statement in the “Confidential to Editor” section, and submit your "Accept" recommendation.

Reviewer #1: All comments have been addressed

Response :

We appreciate the reviewer’s positive assessment. No changes were made to this section of the manuscript.

2. Is the manuscript technically sound, and do the data support the conclusions?

The manuscript must describe a technically sound piece of scientific research with data that supports the conclusions. Experiments must have been conducted rigorously, with appropriate controls, replication, and sample sizes. The conclusions must be drawn appropriately based on the data presented.

Reviewer #1: Yes

Response :

We appreciate the reviewer’s positive assessment. No changes were made to this section of the manuscript.

3. Has the statistical analysis been performed appropriately and rigorously?

Reviewer #1: Yes

Response :

We appreciate the reviewer’s positive assessment. No changes were made to this section of the manuscript.

4. Have the authors made all data underlying the findings in their manuscript fully available?

The PLOS Data policy requires authors to make all data underlying the findings described in their manuscript fully available without restriction, with rare exception (please refer to the Data Availability Statement in the manuscript PDF file). The data should be provided as part of the manuscript or its supporting information, or deposited to a public repository. For example, in addition to summary statistics, the data points behind means, medians and variance measures should be available. If there are restrictions on publicly sharing data—e.g. participant privacy or use of data from a third party—those must be specified.

Reviewer #1: Yes

Response :

We appreciate the reviewer’s positive assessment. No changes were made to this section of the manuscript.

5. Is the manuscript presented in an intelligible fashion and written in standard English?

PLOS ONE does not copyedit accepted manuscripts, so the language in submitted articles must be clear, correct, and unambiguous. Any typographical or grammatical errors should be corrected at revision, so please note any specific errors here.

Reviewer #1: Yes

Response :

We appreciate the reviewer’s positive assessment. No changes were made to this section of the manuscript.

6. Review Comments to the Author

Please use the space provided to explain your answers to the questions above. You may also include additional comments for the author, including concerns about dual publication, research ethics, or publication ethics. (Please upload your review as an attachment if it exceeds 20,000 characters)

Reviewer #1: Overall, the revision substantially improves transparency and model comparison. However, several consistency fixes and editorial corrections are still needed before acceptance.

For example:

• Duplicate entry — Radečić (items 24 and 32) appears twice; please deduplicate.

• Source quality — Replace non‑scholarly/tutorial sources (e.g., Medium, Analytics Vidhya) with primary documentation or peer‑reviewed publications wherever possible.

Response :

We appreciate the reviewer’s constructive comments. We carefully reviewed the reference list by updated them with scholarly source and removed those duplication references as follows:

1. To improve source quality, non-scholarly/tutorial sources were replaced with peer-reviewed primary references:

1.1 Reference [24] was replaced with: Bergmeir C, Hyndman RJ, Koo B. A note on the validity of cross-validation for evaluating autoregressive time series prediction. Comput Stat Data Anal. 2018;120:70–83. https://doi.org/10.1016/j.csda.2017.11.003

1.2 Reference [25] was replaced with: Chen T, Guestrin C. XGBoost: A scalable tree boosting system. Proc 22nd ACM SIGKDD Int Conf Knowledge Discovery and Data Mining. 2016:785–794. https://doi.org/10.1145/2939672.2939785

2. Those non-scholarly and duplicate references were removed:

2.1 Former Reference [32] has been removed.

2.2 Former Reference [33] has been removed

3. The in-text citations in Section 3 (Results and Discussion) were updated accordingly, with former references [32, 33] has been replaced with [24, 25].

7. PLOS authors have the option to publish the peer review history of their article ([what does this mean?](https://track.editorialmanager.com/CL0/https:%2F%2Fjournals.plos.org%2Fplosone%2Fs%2Feditorial-and-peer-review-process%23loc-peer-review-history/1/010f019cada8f471-50d9c49a-d5d2-4d81-ad93-cd4effccd5ea-000000/_T6LVXqqd2omLxCGxuWApd6-jmsIeoRVk8LYFKrD2JM=252)). If published, this will include your full peer review and any attached files.

If you choose “no”, your identity will remain anonymous but your review may still be made public.
Do you want your identity to be public for this peer review? For information about this choice, including consent withdrawal, please see our [Privacy Policy](https://track.editorialmanager.com/CL0/https:%2F%2Fwww.plos.org%2Fprivacy-policy/1/010f019cada8f471-50d9c49a-d5d2-4d81-ad93-cd4effccd5ea-000000/0wNi6oFg_6jqDFqccXvq_dy2SNeGpWwJ3uANZz_8Wbs=252).

Reviewer #1: No

Response :

Not applicable. This comment concerns reviewer privacy preferences and does not require manuscript modification.
